# You Look Good Enough to Eat: A Brief Exploration of Human Cannibalism and Mental Illness

**DOI:** 10.1192/j.eurpsy.2023.2040

**Published:** 2023-07-19

**Authors:** S. Jesus, A. R. Costa, G. Simões, A. I. Gomes, P. Garrido

**Affiliations:** Departamento de Psiquiatria e Saúde Mental, Centro Hospitalar do Baixo Vouga, Aveiro, Portugal

## Abstract

**Introduction:**

Although evidence of cannibalism in humans dates back millennia, for most civilized societies, it is an unthinkable act of violence and strictly taboo. It is commonly relegated to the domain of horror films and literature, often associated with the likes of Jeffrey Dahmer or Hannibal Lecter. However, for some, this theme encompasses a pathological or sexual realm. Vorarephilia or sexual cannibalism is, at its simplest level, a psychosexual disorder characterized by the erotic desire to be consumed by, or to personally consume, another human being´s flesh.

**Objectives:**

The authors aim to review human sexual cannibalism as a concept and its eventual relationship to mental illness with recourse to the description of cases of human cannibalism documented in the literature.

**Methods:**

A brief non-systematized literature review utilizing various databases including *Pubmed* and *Google Scholar*, as well as complimentary literature and case reports when pertinent to the theme was performed.

**Results:**

Although cannibalism is a common phenomenon in the animal kingdom, its expression in humans is assumed to be a minority occurrence and relegated to stories of a more primal past. Pathological cannibalism is an extremely rare occurrence and has been described in association with severe psychotic mental illness and extreme forms of significant paraphilia. Sexual cannibalism appears as a rarity in humans and although the majority with this paraphilia do not partake in actual human consumption, remaining a fantasy-based desire, cases of cannibalism have been reported and tried.

**Conclusions:**

Eating the flesh of one’s own species is probably one of the few remaining taboos in modern human societies. In humans, cannibalism is a rare occurrence and has been associated with mental illness. Due to the rarity of this phenomenon, with few cases documented in the literature, the underlying etiology, as well as potential environmental and individual risk factors are still to be defined, indicating a potential for further study.

**Disclosure of Interest:**

None Declared

